# Extracellular Xylanopectinolytic Enzymes by* Bacillus subtilis* ADI1 from EFB's Compost

**DOI:** 10.1155/2017/7831954

**Published:** 2017-04-24

**Authors:** Muhammad Hariadi Nawawi, Rosfarizan Mohamad, Paridah Md. Tahir, Wan Zuhainis Saad

**Affiliations:** ^1^Faculty of Biotechnology and Biomolecular Sciences, Universiti Putra Malaysia (UPM), 43400 Serdang, Selangor, Malaysia; ^2^Institute of Tropical Forestry and Forest Products, Universiti Putra Malaysia (UPM), 43400 Serdang, Selangor, Malaysia

## Abstract

Microbial xylanase and pectinase are two extremely valuable enzymes, which have captivated much attention. This can be seen from the increased demand for these enzymes by many industrial sectors. This study investigates the isolation and screening of extracellular xylanopectinolytic enzymes-producing bacteria in a submerged fermentation (SmF). Samples are collected from the compost of empty fruit bunch (EFB) at Biocompost Pilot Plant, located at Biorefinery Plant, Universiti Putra Malaysia. From the experiment, out of 20 isolates, 11 isolates show xylanase or/and pectinase activity, and only one isolate (EFB-11) shows the concurrent activities of xylanase and pectinase. These activities are selected for enzyme production under submerged fermentation (quantitative screening). At the 72nd hour of incubation, xylanase and pectinase show the highest production, which ranges about 42.33 U/mL and 62.17 U/mL (with low amount of cellulase present), supplemented with 2% (w/v) of rice bran as carbon source at incubation temperature level, which is 30°C. Meanwhile, the pH of media is shifted to 8.42, which indicates that EFB-11 isolate is alkalotolerant bacteria and identified as* Bacillus subtilis* ADI1. This strain proves to have potential in agroindustrial bioconversion and has a promising ability to scale up to an industrial scale.

## 1. Introduction

“Environment-friendly” technologies are demanded for industrial processes. The use of enzymes, especially, is gaining momentum in several frontier developments. Furthermore, xylanase and pectinase are the enzymes which are vital in the industrial sector, and they are widely used in the paper and pulp industry, animal feed, textile industry, oil extraction, tea and coffee fermentation, waste paper recycling, and the fruit juice industry [1–5].

One of the potential applications of xylanolytic and pectinolytic enzyme system is found in the pulp and paper industry, in which their enzymatic actions are involved in the removal of xylan and pectin from kraft pulp fiber. This consequently increases the brightness of pulp [6–8]. Moreover, the pectin in the plant cambium cells constitutes up to 40% of the plant cambium dry weight. This makes the pectinase play a main role in degrading the available pectin. Other than that, xylanase may also be involved in degradation, since the phloem of cambium cells contains a high amount of hemicellulose [9, 10].

In order to degrade pectin on the pulp fibers, the treatment of pectinase is required, in order to weaken the chemical bond between cellulose and lignin before their further refinement during chemical bleaching. Pulp-treated pectinases introduce the separation of pulp microfibrils and pulp fibers, compared to the smooth surfaces of untreated pulp. Additionally, they also promote efficient loosening and swelling of pulp fibers, greater porosity, and loss in the compactness of the pulp fibers, when observed under scanning electron microscope (SEM). This indicates that only a low amount of chlorine compound is required, since enzymatic treatment renders the pulp fibers more accessible to chemical bleaching [11]. In addition, high cationic demand of pectins results in the yellowness of paper and weakens the dewatering during sheet formation. These outcomes are also caused by pectinases; pectinases depolymerize polygalacturonic acid in the pulp, as they consequently decrease the cationic demand in the filtrate from peroxide bleaching [12].

The replacement of conventional pulp bleaching could become a new fiber liberation technology in pulp and paper industry. This is because it uses high amount of chemical additives. Besides that, the additives involve the use of enzymatic bleaching, which is ecofriendly and cost-effective and has a product yield. Both xylanases and pectinases play an important role in the bleaching practice, which is the earliest step in the process of papermaking [10, 13].

In general, bacterial-producing xylanopectinolytic enzymes can be found in environments rich in lignocellulosic and hemicellulosic material as their microbial sources. Examples of microbial sources are soil-enriched compost area, rotten and decayed wood, and agroresidual wastes [14]. Therefore, the objective of the present study is to isolate xylanopectinolytic enzymes-producing bacteria from plant compost.

## 2. Materials and Methods 

### 2.1. Isolation of Bacteria from Compost Area

Samples were collected from the compost of empty fruit bunch (EFB) at Biocompost Pilot Plant, which was located at Biorefinery Plant, Universiti Putra Malaysia, with surrounding temperature of 30°C.

A tenfold serial dilution (10^1^ to 10^7^ dilutions) was performed in 10 mL scale. 1 mL of each sample of the dilution was added into 9 mL of sterile 0.85% (w/v) NaCl solution, and it was serially transferred before getting thoroughly mixed. The amount of each serial dilution was 0.1 mL aliquots, and it was spread on a plate using hockey stick on sterile nutrient agar. Then, the dilution was incubated for 24 hours at a temperature of 30°C [15]. NA plates were prepared and autoclaved at 121°C, 15 psi, and 15 min. NA was used for growing and purifying the bacteria culture. In order to obtain a pure culture, grown bacterial colonies were observed on plates, and the colonies were subcultured several times to obtain single colonies. Pure culture was verified using Gram's stain. The bacteria isolated were maintained on agar slants and incubated at 4°C with subculturing every four to six weeks. Meanwhile, stock culture was preserved in freeze-dried form in an ampoule, which was stored at −20°C in 15% glycerol.

### 2.2. Primary Screening of Xylanopectinolytic Enzymes-Producing Bacteria

Primary screening was conducted on xylan agar medium and pectin agar medium. Basal agar medium was used for the screening of xylanopectinolytic enzymes-producing bacteria (g/L: peptone, 5; yeast extract, 5; MgSO_4_·7H_2_O, 0.2; K_2_HPO_4_, 1; agar, 15; pH 7) supplemented with 1% of beechwood xylan in order to prepare xylan agar medium (used for screening xylanase-producing bacteria), as described by [16]. Pectin agar medium was used to screen pectinase-producing bacteria by replacing beechwood xylan with pectin from citrus peel [17]. The Na2CO3 was used to adjust the pH of all the media, and it was sterilized separately.

Bacterial suspension (grown 24 hours in nutrient broth) samples (100 *μ*L) were loaded in the wells of xylan agar medium (for xylanase producers) and pectin agar medium (for pectinase producers). Then, a sterile (diameter, 10 mm) borer was used to create wells in solidified plates, and the plates were incubated at 30°C (based on surrounding temperature during sampling) for 24 hours [18, 19]).

The plates were flooded with Gram's iodine solution [20]. Positive control (*Bacillus subtilis* ATCC 6633) was used as a comparison in determination of clear zone [17, 21, 22]. The positive strains which exhibited clearance zone around the colony were selected for secondary screening in submerged fermentation (liquid medium) and maintained on nutrient agar slant at 4°C.

### 2.3. Secondary Screening of Xylanopectinolytic Enzymes-Producing Bacteria

Following primary screening, the bacteria cultures were further narrowed down by secondary screening in submerged fermentation. Substrate (rice bran) was used as the main carbon source, while other components of media (basal medium) remained the same.

In secondary screening, the isolates were carried out in 250 mL Erlenmeyer flasks containing 50 mL of basal medium. The basal medium was supplemented with 1% (w/v) of rice bran and incubated with 1% (v/v) of inoculums (18 h old, OD ~ 0.85), subjected to shaking condition (200 rpm) for a period of 144 hours at ambient temperature (30°C). Crude xylanopectinolytic enzymes were harvested every 25 hours, and they were used for the measurement of pH and the optical density of bacterial growth. Meanwhile, the cell supernatant (treated as crude enzyme) was used for xylanase, pectinase, and cellulose assay, protein estimation, and reducing sugar quantification. The secondary screening studies were tested in triplicate.

### 2.4. Analytical Methods

All the analytical methods were performed including the determination of enzymes assay, reducing sugar, and soluble protein as described by Bradford (1976).

### 2.5. Enzymes Assay for Xylanase, Pectinase, and Cellulase Enzymes

The enzyme activities were measured according to the method described by Miller (1959). Substrates used in assaying activity of xylanase, pectinase, and cellulase were 1% (w/v) of beechwood xylan, 0.5% (w/v) of polygalacturonic acid, and 1% (w/v) of carboxymethyl cellulose, respectively. The reaction mixture which was 490 *μ*L of 0.01 M glycine NaOH buffer (pH 8.5) containing respective substrate was added to the test tube followed by the addition of 10 *μ*L of appropriately diluted crude enzyme (culture filtrate) as proposed by Kaur et al. [23]. Then, the reaction mixture was incubated at 55°C for 10 min. This is followed by the addition of 1.5 mL of dinitrosalicyclic acid reagent, referred to as DNS, in order to stop the reaction.

Enzyme activity is expressed in one unit and defined as the release of one *μ*mol of xylose, galacturonic acid, and glucose, respectively. It was liberated per mL enzyme per min under the assay conditions (U/mL).

### 2.6. Molecular Identification Using 16S rDNA Sequencing

The selected isolate was grown in nutrient broth for 24 hours at 30°C, with shaking at 200 rpm. The extraction of DNA was done by using G-spin™ Genomic DNA Extraction Kit, provided by iNtRON Biotechnology, Korea. The extracted genomic DNA was purified using FavorPrep™ Genomic DNA Mini Kit (Favorgen Biotech Corp., Taiwan). A 16S rDNA fragment was amplified using i-Taq™ plus DNA Polymerase (iNtRON Biotechnology, Korea) with the universal primers 27F (5′-AGAGTTTGATCMTGGCTCAG-3′) and 1492R (5′-TACGGTTACCTTGTTACGACTT-3′) [24]. The PCR products were sent to a private laboratory (NHK Bioscience Solutions Sdn. Bhd., Kuala Lumpur, Malaysia) for sequencing. The sequence data was deposited in NCBI (National Centre for Biotechnology Information, https://www.ncbi.nlm.nih.gov/) GenBank and also used for BLAST (Basic Local Alignment Search Tool) analysis.

## 3. Results and Discussion

### 3.1. Isolation of Bacteria from Compost Area

The soil samples collected in this study consisted of soil from the compost (decayed organic materials) in Biocompost Pilot Plant, which was located at Biorefinery Plant, Universiti Putra Malaysia, Serdang, Malaysia.

In this study, the soil samples near compost sites were targeted because the xylanase and pectinase-producing microbes have been frequently reported to be isolated from the agricultural wastes and soils. These are where the hemicellulose materials were deposited. Besides that, the compost sites selection was also due to the nutrient rich environments [17]. Previously, xylanopectinolytic enzymes producers have been isolated from soils, as stated by Kaur et al. [25] and Ahlawat et al. [21].

The cultures were grown on nutrient medium and 20 isolates, with distinctive macroscopic morphology being selected and preceded with Gram's stain. This was to verify the purity of a culture, instead of determining the Gram-negative or Gram-positive bacteria and their cellular morphologies. The cultural characteristics of 20 isolates are shown in Table 1.

EFB-11 is a rod-shaped Gram-positive bacteria with a violet color of Gram's stain. Based on the microscopic view of Gram's stain slide, the constant identical features of bacteria's shapes and colors demonstrated that the pure colony of EFB-11 was obtained from isolation techniques.

### 3.2. Screening of Xylanopectinolytic Enzymes-Producing Bacteria

Twenty bacteria isolates were successfully inoculated. The primary screening of xylanopectinolytic enzymes-producing bacteria was performed and resulted in the formation of clearance zone on xylan and pectin containing agar. The primary screening, which resulted in 11 isolates, displayed the zone of clearance on either xylan agar medium or pectin agar medium. However, only one isolate showed the concurrent reaction of zone clearance in xylan and pectin agar medium (Table 2). That isolate was selected for secondary screening, in order to determine the quantitative analysis on the enzymes produced.

EFB-11 displayed higher clearance zone diameter for xylanase and pectinase (25 mm and 36 mm of size, resp.), as compared to the control-type strain* Bacillus subtilis* ATCC 6633 (21 mm and 32 mm of size). The formation of large clearance zone, which was around the growth of an isolate on solid agar plates, demonstrated the presence of the membrane-bound hydrolase with good substrate hydrolysis capability [17, 26]. Therefore, the hypothesis that the growing colony resulted in the clear zone formation was possibly because of the regional presence of enzymes secreted near the colony.

Even so, this result was not fully expressed during submerged fermentation (SmF) cultivation [27, 28]. Therefore, EFB-11 was subjected to liquid culture cultivation (SmF), using rice bran as a carbon source. This provided a clear understanding of xylanase and pectinase producer. As mentioned by Kaur et al. [23], the culture was grown in liquid medium, with some modifications supplemented with rice bran at 1.0% (w/v) level as carbon source. Then, EFB-11 was grown in fermentation medium for 144 hours.

Culture broth's pH was measured at every 24-hour interval, and the clear supernatant was used for the estimation of enzymes' activity [29], soluble protein [30], and reducing sugar [29]. In this study, submerged fermentation (SmF) technique was chosen, generally because bacteria require high moisture content to grow. Therefore, SmF was the only technique that implemented free-flowing liquid substrates in its systems, compared to solid state fermentation (SSF) [31]. The data of growth profile and enzymes production for EFB-11 is shown in Table 3.

Catabolite repression on enzymes production apparently occurred at the hour of maximum enzyme production, which resulted in lowering of the reducing sugar levels. There are several reports regarding the catabolite repression of xylanase [32–35] and pectinase [36–39] in bacteria and fungi.

After 72 hours of growth, the amount of xylanase and pectinase activity began to slightly decline. It was possible that these enzymes were digested by protease released by the autolysis of cells [40–42]. During the early hours of growth (log phase), reducing sugar concentration showed steep increase, presumably due to the fact that xylan and pectin in the medium were hydrolyzed by xylanase and pectinase, which were present in considerable amount in the inoculum [43].

During fermentation period, protein concentration did not fluctuate much in the culture medium. However, it slightly declined after 120 hours of fermentation period, while the pH of medium shifted towards alkaline values by 144 hours of growth. Compared to any other industrial applications, especially those related to pulp and paper industries, the usage of alkaline pH enzymes is preferable and very important properties compared to fungal enzymes, that is, slightly acidic, require extra processes in subsequent steps. This causes them to be less suitable for practical applications, as the requirement of its cost also increases. The presence of cellulase in preparation of crude enzyme could affect cellulose fibers. The negative effects on cellulase could destroy the cellulose structure, which results in serious viscosity drop of pulp due to cellulose hydrolysis. Consequently, paper quality will possibly be diminished [44, 45].

Since EFB-11 was* Bacillus* sp. with maximum activity 42.33 U/mL (xylanase) and 62.17 U/mL (pectinase), it was found to be a comparable with reported cases. Earlier reports regarding* Bacillus* sp. from many researchers are listed as follows: Dhillon et al. [46] reported xylanase activity of 20.6 U/mL. Meanwhile, other reports included the observations performed by Nakamura et al. [47], 10 IU/mL; Dahlberg et al. [48], 3-4 IU/mL; Keskar [49], 70–72 IU/mL; and Zychlinska et al. [50], 70.4 IU/mL. Sunnotel and Nigam [51] reported an activity of pectinase of 30.1 U/mL, and Kashyap et al. [52] observed an activity of 53 U/mL by a* Bacillus* sp. DT7.

Hence, the xylanase and pectinase production, which was displayed by isolate EFB-11, was comparable with the previous reports. Their production could be further increased by the optimization of fermentation conditions.

### 3.3. Identification of Bacterial Culture Using 16S rDNA Gene Sequence Analysis

In NCBI database, BLAST showed significant alignments of top three highest similarities between* Bacillus subtilis* sp. The* Bacillus subtilis* sp. with 98% similarity were* B. subtilis* subsp.* spizizenii* strain ATCC 6633 (NR 112049),* Bacillus subtilis* subsp.* spizizenii* strain NBRC 101239 (NR 112686), and* Bacillus subtilis* subsp.* inaquosorum* strain BGSC 3A28 (NR 104873). Moreover, the isolate EFB-11 was identified as* Bacillus subtilis* strain ADI1. This is because, based on NCBI database, its 16S rDNA gene sequence shows the highest degree of similarity to 16S rRNA gene sequences of* B. subtilis* culture collection reference strains in GenBank.

**Figure 1:**
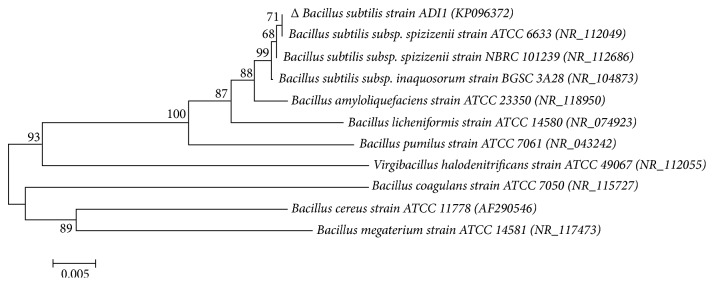
Phylogenetic relationship of (Δ)* Bacillus subtilis* ADI1 with other* Bacillus *sp., based on neighbor-joining method. The bar indicates the number of nucleotide substitutions per site. Bootstrap values were obtained with 1,000 replications and the values are shown at the nodes. Their names and respective accession numbers are shown on the tree.

The construction of a phylogenetic tree (Figure 1) was based on the comparison made on the 16S rDNA sequence of isolate EFB-11 and other strain of* Bacillus *species, with ATCC coded. CLUSTALW from the Biology Workbench database (http://workbench.sdsc.edu) was used in aligning the sequences [53, 54]. GenBank database was used to obtain 16S rDNA's sequences of other* Bacillus* species. The evolutionary phylogenetic tree was generated and analyzed using a software package, which was MEGA6 [55]. The result gathered from the 16S rDNA analyses implied that the* Bacillus subtilis *ADI1 was very close to other* Bacillus subtilis* strains.

## 4. Conclusions 

Agricultural waste or compost (decayed organic materials) area has high populations of microorganisms, and it could serve as a source of microorganisms which produces xylanase and pectinase enzymes. These results indicated that xylanopectinolytic enzymes-producing microorganism can also be screened in different environments. The screening of the total of 20 isolates from the compost resulted in 11 isolates with xylanase or pectinase enzyme-producing bacteria. From the 11 isolates, only one isolate was found to be xylanase and pectinase enzymes producer. The mentioned isolate was identified as* Bacillus subtilis* ADI1. The bacterium* Bacillus subtilis* ADI1 was selected as being active in xylan and pectin hydrolysis, and it is a potentially high extracellular xylanopectinolytic enzymes producer for further optimization of enzymatic production studies.

## Figures and Tables

**Table 1:** Morphological characteristics of isolates.

Isolate number	Cultural characteristics	Gram's nature
EFB-1	Flat, circular, entire	Gram-positive, *Bacillus*
EFB-2	Convex, circular, entire	Gram-positive, *Bacillus*
EFB-3	Flat, circular, entire	Gram-positive, *Bacillus*
EFB-4	Convex, irregular, entire	Gram-positive, *Bacillus*
EFB-5	Convex, circular, undulate	Gram-positive, *Bacillus*
EFB-6	Flat, irregular, entire	Gram-negative, *Bacillus*
EFB-7	Convex, irregular, entire	Gram-positive, *Bacillus*
EFB-8	Convex, circular, entire	Gram-positive, *Bacillus*
EFB-9	Convex, irregular, entire	Gram-negative, coccus
EFB-10	Convex, irregular, undulate	Gram-negative, coccus
EFB-11	Umbonate, circular, entire	Gram-positive, *Bacillus*
EFB-12	Convex, circular, entire	Gram-negative, coccus
EFB-13	Convex, irregular, entire	Gram-positive, *Bacillus*
EFB-14	Convex, circular, entire	Gram-positive, *Bacillus*
EFB-15	Flat, irregular, entire	Gram-negative, *Bacillus*
EFB-16	Umbonate, circular, entire	Gram-positive, *Bacillus*
EFB-17	Convex, circular, entire	Gram-negative, coccus
EFB-18	Convex, circular, undulate	Gram-negative, coccus
EFB-19	Convex, circular, entire	Gram-positive, *Bacillus*
EFB-20	Flat, irregular, undulate	Gram-positive, *Bacillus*

**Table 2:** Primary screening of selected isolates on agar plate with clear zone diameter.

Isolate number	Clear zone size (mm)	
	Xylan agar	Pectin agar
EFB-1	nd	14
EFB-6	nd	15
EFB-9	nd	14
EFB-12	nd	13
EFB-15	nd	14
EFB-3	nd	22
EFB-8	17	nd
EFB-9	13	nd
EFB-14	16	nd
EFB-19	16	nd
EFB-11	25	36
^*∗*^ *B. subtilis* ATCC 6633	21	32

^*∗*^Positive control strain; nd: not detected.

**Table 3:** Growth profile and enzymes production by EFB-11 from initial screening.

Period of maximum enzyme production (hours)	Optical density	pH	Composition and enzymes activity of cell-free supernatant harvested at the hour of maximum enzyme production				
			Soluble protein (mg/mL)	Reducing sugar (ug/mL)	Xylanase activity (U/mL)	Pectinase activity (U/mL)	Cellulase activity (U/mL)
0	0.000 ± 0.001^a^	7.00 ± 0.01^a^	0.200 ± 0.010^a^	2.50 ± 0.001^a^	0.03 ± 0.001^a^	0.01 ± 0.001^a^	0.00 ± 0.001^a^
24	0.550 ± 0.019^b^	7.47 ± 0.05^b^	0.203 ± 0.030^a^	2.69 ± 0.006^b^	22.33 ± 0.050^b^	37.33 ± 0.115^b^	1.33 ± 0.005^b^
48	0.670 ± 0.077^c^	8.12 ± 0.05^c^	0.251 ± 0.019^b^	3.39 ± 0.002^c^	39.00 ± 0.100^c^	41.67 ± 0.289^c^	1.47 ± 0.05^c^
72	1.257 ± 0.001^d^	8.42 ± 0.03^d^	0.368 ± 0.018^c^	3.90 ± 0.001^d^	42.33 ± 0.150^d^	62.17 ± 0.029^d^	1.47 ± 0.012^c^
96	1.110 ± 0.006^e^	8.57 ± 0.14^e^	0.580 ± 0.018^d^	3.90 ± 0.001^d^	40.00 ± 0.001^e^	60.17 ± 0.029^e^	1.80 ± 0.005^d^
120	1.000 ± 0.029^f^	8.50 ± 0.01^ed^	0.332 ± 0.020^e^	2.99 ± 0.001^e^	27.00 ± 0.100^f^	47.90 ± 0.364^f^	1.41 ± 0.003^e^
144	0.932 ± 0.001^f^	8.40 ± 0.01^d^	0.313 ± 0.022^e^	2.69 ± 0.001^b^	27.00 ± 0.100^f^	52.17 ± 0.029^g^	1.31 ± 0.003^b^

Values are mean of triplicate ± standard deviation.

a–g Means values in same column with different superscripts are significantly different (*P* < 0.05).

## Abbreviations

SmF: Submerged fermentation
SSF: Solid state fermentation
EFB: Empty fruit bunch
*B*.: * Bacillus*.
